# The potential investment impact of improved access to accelerated approval on the development of treatments for low prevalence rare diseases

**DOI:** 10.1186/1750-1172-6-49

**Published:** 2011-07-06

**Authors:** Brigitta E Miyamoto, Emil D Kakkis

**Affiliations:** 1 Kakkis EveryLife Foundation For Rare Diseases, 77 Digital Drive, Suite 210, Novato, CA 94949, USA

**Keywords:** Accelerated approval, Rare diseases, Drug development, Surrogate endpoint, Clinical trials

## Abstract

**Background:**

Over 95% of rare diseases lack treatments despite many successful treatment studies in animal models. To improve access to treatments, the Accelerated Approval (AA) regulations were implemented allowing the use of surrogate endpoints to achieve drug approval and accelerate development of life-saving therapies. Many rare diseases have not utilized AA due to the difficulty in gaining acceptance of novel surrogate endpoints in untreated rare diseases.

**Methods:**

To assess the potential impact of improved AA accessibility, we devised clinical development programs using proposed clinical or surrogate endpoints for fifteen rare disease treatments.

**Results:**

We demonstrate that better AA access could reduce development costs by approximately 60%, increase investment value, and foster development of three times as many rare disease drugs for the same investment.

**Conclusion:**

Our research brings attention to the need for well-defined and practical qualification criteria for the use of surrogate endpoints to allow more access to the AA approval pathway in clinical trials for rare diseases.

## Introduction

Patients with rare diseases have had difficulty obtaining disease-specific treatments due to inadequate biotechnology investment despite the existence of promising science. The 1983 Orphan Drug Act was passed to address this problem, offering financial incentives to companies developing rare disease drugs. During the first 25 years since the passage of the Act, 1,892 drugs were given orphan designation, 326 of which have been approved [1]. While this astounding success has significantly impacted rare disease-affected patients, 95% of over 6,000 rare diseases still have no specific treatment.

Although there are numerous challenges in development of a disease treatment, the most critical is the clinical study process. By law, all drugs must undergo clinical trial testing to demonstrate safety and substantial efficacy before FDA approval. This process usually requires a Phase 3, double-blind, placebo-controlled trial, widely regarded as the "gold standard." Phase 3 trials typically assess efficacy using a measure of clinical benefit such as how a patient feels, functions, or survives [2]. For disorders with very few and variable patients or lengthy time courses or irreversible disease progression, the use of clinical measures as endpoints can make specific treatment development intractable for practical and ethical reasons.

The Accelerated Approval (AA) regulations were promulgated by FDA in 1992 to drive the development of new treatments for serious and life-threatening disorders, primarily motivated by the AIDS crisis and the slow pace of treatment development for HIV infection [3]. In HIV, monitoring death rate or complex endpoints such as hospitalization for opportunistic infections was difficult or unethical. Consequently, AA regulations allowed for drug approval based on the use of surrogate endpoints "reasonably likely to predict clinical benefit" [3] - a surrogate endpoint being a measure, such as a blood test or urine marker, believed to be indicative of a disease state and treatment effect, but not demonstrative of a direct health gain to the patient.

AA has been enormously successful at driving innovation in the development of cancer and HIV therapies during the first 16 years: 26 new chemical entity (NCE) cancer drugs have been approved, using either tumor load or progression-free survival as surrogate endpoints, and 29 HIV drugs (25 NCEs and 4 combination drugs) have gained approval using either CD4 count or viral load as endpoints [4]. In the case of HIV, using survival as an endpoint would have made drug trials too time-consuming due to the lengthy disease course, too costly due to the high number of required patients, and unethical due to the use of placebo in a lethal condition. Even more critical for the development of HIV treatment was the ability to test the complex combinations of drugs responsible for the substantial improvement in long-term outcome. Drug combination studies would have been impossible using a clinical endpoint given large patient numbers and extended study lengths, despite the fact that drug combinations are now essential to the HIV treatment process. Current HIV therapies are a prime example whereby great scientific ideas would nonetheless have led to little or no treatment success without AA access. These successes show that the AA regulatory pathway is having a profound impact on innovation in treatment development.

Rare and ultra-rare diseases have not shared in cancer and HIV's successes. Only one drug among the 73 NCEs approved under AA, agalsidase beta (Fabrazyme ^®^), has been used to specifically treat a rare genetic disease, Fabry [4]. Although treatments for biochemical genetic diseases, such as urea cycle disorders and phenylketonuria (PKU), have been approved through traditional FDA pathways using blood test endpoint-driven trials, the existence of regulatory precedents for approval (urea cycle drugs), substantial routine disease management history, and published study data (PKU drugs), supported the use of blood test-based endpoints [5,6]. Most ultra-rare diseases do not have the same pre-existing body of clinical management or historical study data currently required to utilize the AA pathway. For example, during the clinical development of Aldurazyme to treat mucopolysaccharidosis I (MPS I), there was insufficient independent clinical data to support the use of the surrogate endpoint of urine glycosaminoglycan excretion to predict clinical benefit despite substantial scientific, animal, and clinical data supporting its relevance acquired in clinical trials. A second study using clinical endpoints was required for approval [7,8]. With this essential requirement for independent clinical data, the AA pathway is virtually unavailable for novel drugs developed for untreated ultra-rare diseases.

The European Medicines Agency grants Conditional Marketing Authorization (CMA), similar to AA, to disease treatments addressing unmet medical need, including orphan diseases. Under CMA guidelines, treatments are conditionally approved on the basis of a presumed positive benefit-risk profile, and further confirmatory studies are agreed upon to establish clinical benefit. While the use of surrogate endpoints to obtain a CMA is not explicitly mentioned in legislation, industry has demonstrated a willingness to cooperate with government to make this a reality, and the necessity of collaboration between academia, government, and industry has been foreseen [9,10]. Therefore, the use of surrogate endpoints to achieve drug approval is a pressing issue in more than one continent.

Three factors influencing the likelihood of initiation of a development program for rare disease treatment have been evaluated recently: prevalence, disease class, and scientific output. It is less than one-third as likely that a drug treating an ultra-rare disease with a very low prevalence of 0.1 to 9 per 100,000 enters the development process, as compared to a rare disease having a prevalence of 10 to 50 per 100,000 [11]. The authors interpreted their finding as an indication that additional economic incentives and initiatives are necessary to promote ultra-rare disease treatment development.

We believe improving access to the AA pathway could potentially fulfill this need. By studying the effect of increased access to AA for ultra-rare diseases of a specified disease class and having ample scientific output, our analysis isolates one of the three factors studied by the authors: prevalence. We believe that all rare diseases, even those with more limited research activity or more poorly understood, would also benefit from this increased access to the AA pathway, given that a clearer path to approval will then drive more research in more productive directions.

To identify scientifically promising disease treatments that have not translated to human approved use and which might benefit from access to the AA pathway, we searched for ultra-rare disease therapies that consistently reversed disease pathology in animal models, but had stalled in development. We uncovered numerous examples of successful treatments, and investigated the probable effect that access to AA would have on investment potential for their development. After reviewing the diseases and their underlying science, we established reasonable clinical and surrogate endpoints and devised development programs dependent on both endpoint types. We demonstrate that improved AA access could enhance ultra-rare disease treatment feasibility and investment potential by decreasing the development cost to approval, shortening the time to approval, and increasing potential investment return. We are not verifying or implying that these surrogates have been proven to be valid, nor at this time are we suggesting methods to qualify surrogates more efficiently. By highlighting and quantifying the substantial benefit to development that we observe, we hope to spur experts in this field to discuss practical and rational solutions to the challenges of qualifying surrogates as primary endpoints for pivotal clinical trials in ultra-rare diseases.

## Methods

### Literature search

PubMed searches to locate successfully treated animal models included keywords "disease name," "treatment," "enzyme replacement therapy," "specific name of therapy," and "animal model." To confirm lack of FDA drug approval, the Drugs@FDA database was searched by "disease name." Diseases identified are shown in Table 1.

**Table 1:** Fifteen rare diseases with potential treatments.

Disease class		Disease	Approx. patient number	Treatment	Pub. year
Lysosomal storage		α-Mannosidosis	200	i.v. α-mannosidase	2004
		Aspartylglucosaminuria	400	i.v. glycosyl- asparaginase	2000
		Galactosialidosis	100	i.v. PPCA	2004
		Mucopolysaccharidosis IV A (MPS IVA)	2,000	i.v. GALNS	2008
		Mucopolysaccharidosis VII (MPS VII)	200	i.v. β-glucuronidase	1994
	Neurological	GM1 Gangliosidosis	850	oral chaperone N-octyl-4-epi-β-valienamine	2003
		Late Infatile Neuronal Ceroid Lipofuscinosis (LINCL)	600	intraventricular TPP1	2008
		Metachromatic Leukodystrophy (MLD)	4,000	intrathecal ASA	2005
		Mucopolysaccharidosis IIIA (MPS IIIA)	1,300	intra-CSF sulfamidase	2004
		Niemann-Pick B	650	i.v. ASM	2000
		Lysosomal Acid Lipase Deficiency (LAL Deficiency)	150	i.v. mannose-6-phosphate terminated LAL	2001
Enzyme deficiencies affecting:	Kidneys	Primary Hyperoxaluria	2,400	oral crystalline oxalate-decarboxylase	1999
	Skin and connective tissue	Recessive Dystrophic Epidermolysis Bullosa (RDEB)	500	i.d. C7	2004
		X-Linked Hypohidrotic Ectodermal Dysplasia (X-Linked HED)	700	i.v. EDA1	2003
	Carb. metabolism	Congenital Disorder of Glycosylation Ib (CDG-Ib)	100	oral mannose	1998

The table shows the approximate patient number and treatments, based on literature reviewed and author expertise. Specific disease data obtained from published articles, references 26, 43-56. Approximate patient number calculation using OMMBID and Orpha.net data, as described in Methods. Eleven of the 15 diseases represent lysosomal storage disorders. The other four are diverse enzyme deficiencies affecting the kidneys, skin and connective tissue, and carbohydrate metabolism. Proposed treatments for the 15 diseases include protein or substrate replacement, chaperone therapy, metabolic diversion of an accumulated toxic compound, and enzyme replacement therapies (ERTs).

### Endpoints

All chosen endpoint values are located in Table 2. The 6 minute walk test (6MWT) was selected as clinical endpoint in the majority of lysosomal storage disorders (LSDs) exhibiting a varied combination of symptoms. The Mullen Scales of Early Learning (MSEL) was chosen as clinical endpoint for the neurological LSDs. An exception was the selection of the modified Hamburg LINCL scale, specifically designed to measure neurological function in patients with LINCL, which has been used in the determination of gene therapy efficacy in this disease [12]. 6MWT and MSEL data is not readily available for diseases not yet in clinical trials, and thus predicted 6MWT values were obtained from the Phase 3 MPS I laronidase study [13], and predicted MSEL values from a study of bone marrow transplants in Hurler patients (personal communication, Dr. Elsa Shapiro).

**Table 2:** Parameters used in NPV and cost to approval calculations.

		Clinical					Surrogate				
**Disease**	**Estimated cost of treatment**	**Endpoint**	**Sample size**		**Time to see effect**		**Endpoint**	**Sample size**		**Time to see effect**	
					
			**I/II**	**III, IV**	**I/II**			**I/II**	**III**	**I/II**	**III**

α-Mannosidosis	$300,000	6 Minute Walk Test (6MWT) (meters)	12	52	6 mos	6 mos	Urinary Man2GlcNAc (mmol/mol creatinine) measured by HPLC	10	20	3 mos	6 mos
Aspartyl- glycosaminuria	$300,000	6MWT (meters)	12	45	6 mos	6 mos	Urinary aspartylglucosamine (μmol/mmol creatinine)	6	12	3 mos	6 mos
Galactosialidosis	$300,000	6MWT (meters)	12	52	6 mos	6 mos	Urinary oligosaccharides (nmol/mg creatinine)	10	20	3 mos	6 mos
MPS IVA	$300,000	6MWT (meters)	20	52	6 mos	6 mos	Urinary keratan sulfate (ng/g creatinine)	15	30	3 mos	6 mos
MPS VII	$300,000	6MWT (meters)	10	45	6 mos	6 mos	Urinary GAG (μg/mg creatinine)	6	12	3 mos	6 mos
GM1 Gangliosidosis	$120,000	Mullen Scales of Early Learning (MSEL)	20	127	6 mos	1 yr	CSF GM1 ganglioside (pmol/ml)	15	30	3 mos	6 mos
LINCL	$200,000	Modified Hamburg LINCL clinical rating scale	10	30	6 mos	1 yr	CSF neurofilament protein (ng/L)	15	30	3 mos	6 mos
MLD	$200,000	MSEL	20	127	6 mos	1 yr	CSF sulfatide (nmol/L)	10	30	3 mos	6 mos
MPS IIIA	$200,000	MSEL	20	127	6 mos	1 yr	CSF MPS (heparan sulfate)	10	30	3 mos	6 mos
Niemann-Pick B	$300,000	Forced vital capacity (FVC %)	10	30	6 mos	6 mos	Liver size (% change in liver size by MRI)	10	30	6 mos	6 mos
LAL Deficiency	$300,000	Survival	15	30	6 mos	1.5 yrs	Liver size (% change in liver size by MRI)	10	30	6 mos	6 mos
Primary Hyperoxaluria	$300,000	Renal failure	20	183	6 mos	2 yrs	Urinary oxalate (mg/1.73 m²/day)	10	20	3 mos	6 mos
RDEB	$300,000	Hospitalizations	20	61	6 mos	1 yr	Number of anchoring fibrils over 2 × 25 μm of lamina densa	10	30	3 mos	6 mos
X-Linked HED	$100,000	Episodes of severe illness	20	87	6 mos	1 yr	First molar tooth bud presence	10	54	6 mos	6 mos
CDG-Ib	$50,000	Thrombosis	10	30	6 mos	1 yr	Antithrombin III (%)	6	12	6 mos	6 mos

The table shows the diseases, the estimated cost of therapy, and endpoint and study design information; clinical and surrogate sample size and time to see effect values, and is based on references 12-39 and authors' expertise, as described in Methods. Clinical endpoints were chosen from successful development programs (6 minute walk test (6MWT) [13]), or well-accepted clinical problems (renal failure, hospitalizations, or developmental testing), often indicative of a disease's most severe aspect. We chose surrogate endpoints that directly reflected the disease state, and for which the pathologic mechanism was clear; some surrogates were selected for their conceptual link to a chosen clinical endpoint. These surrogates include urinary or CSF substrate accumulation, measures of tissue injury, or alteration in major organs.

Conversely, because urinary and CSF marker information is more readily available even in diseases without treatment, an attempt was made to obtain disease-specific surrogate endpoint values. Urinary and CSF markers are often presented as a "value in patients," and a "value in healthy controls." In this case, when a value in treated patients is unavailable, we estimated the ERT treatment effect to be 60% of total possible improvement. In addition, standard deviations were reduced such that the ratio of standard deviation to surrogate value was maintained as the value of the surrogate decreased. This was done because standard deviations of surrogate markers tend to decrease as heterogeneous patients are treated [13,14]. We obtained information in this manner for α-mannosidosis [15,16], aspartylglucosaminuria [17], galactosialidosis [18], MPS IV A [19], and GM1 gangliosidosis [20]. Urinary GAG values from the Phase 3 MPS I laronidase study [13] were used to estimate the possible effect of ERT in MPS VII. Unpublished data was used to estimate brain injury and sulfatide levels in both late infantile neuronal ceroid lipofuscinosis and metachromatic leukodystrophy. Unpublished values of mean serum pathologic substrate measured during the laronidase Phase 1/2 study obtained from Zacharon Pharmaceuticals were used to estimate treatment effect in MPS IIIA.

To test drug efficacy for Niemann-Pick B, we used % Predicted Forced Vital Capacity [13] as a clinical endpoint since impaired lung function and frequent lung infections are common in this disease. Niemann-Pick B patients experience hepatosplenomegaly which can affect breathing because the large organs infringe upon space normally inhabited by the lungs. Liver size [21] was therefore chosen as a potential surrogate endpoint.

In some diseases, clinical endpoints were chosen to reflect a severe problem faced by patients, namely, survival (LAL Deficiency [22]), renal failure (Primary Hyperoxaluria [23]), hospitalizations (RDEB [24] and X-Linked HED [25]), and episodes of thrombosis (CDG-Ib [26-30]). Surrogate endpoints were almost always chosen to relate directly to disease mechanism, and were often connected to the clinical endpoint: in Primary Hyperoxaluria, urinary oxalate [31,32] increases risk of renal stones, which in turn raises risk of renal failure; and in Congenital Disorder of Glycosylation Ib, episodes of thrombosis are brought about by a deficiency in antithrombin III [33-36] levels. Liver size [21] was chosen as surrogate endpoint for LAL Deficiency. Number of anchoring fibrils in biopsy was chosen as surrogate endpoint for RDEB [37].

In X-linked Hypohidrotic Ectodermal Dysplasia [38], the presence of the first molar tooth bud was chosen as a surrogate endpoint. In the treated canine model [39], the appearance and number of primary teeth were unchanged from the untreated model. However, permanent teeth showed dramatic improvement. We postulated that the same phenomenon might hold true in the treatment of the human disease and chose to measure the appearance of permanent teeth. Evidence of permanent first molar tooth buds, the earliest of human permanent teeth, can be detected by X-ray as early as 6 months after birth.

### Minimum and maximum sample sizes and trial lengths

In order to assure the clinical programs had sufficient safety exposure, a minimum number of patients exposed was set, particularly in the case of very low patient prevalence, or if the treatment effect is expected to be dramatic and need too few study patients. Length and sample size during Phase 3 were primarily determined by time needed to detect efficacy and sample size calculations.

Being an exploratory study, Phase 1/2 trial patient numbers were all set between 6 and 20. The small number of 6 patients was only assigned in the surrogate endpoint Phase 1/2 trial for diseases with extremely low prevalence (Aspartylglycosaminuria, MPS VII, and CDG 1b). The larger number of 20 patients was assigned to those diseases in which the clinical endpoint required over 60 patients in order to detect efficacy in the Phase 3 trial. Ample time was allotted to establish dosing and get a better sense of efficacy.

Patient numbers in Phase 3 trials were primarily determined by sample size calculations. When sample size needed was low, a minimum of 12 patients for a urinary marker, and 30 for any other surrogate or clinical marker, was set. Again, the small number of 12 was reserved for the rarest of diseases. A minimum of 6 months was chosen for a Phase 3 trial to provide adequate safety and exposure information. See Table 2 for trial times and numbers of patients.

### NPV and cost to approval calculation

NPV was calculated by using a template obtained from *Nature Biotechnology *[40], which was then modified to account for a Phase 4 trial in surrogate endpoint development models. For simplicity, the orphan drug tax credit was omitted and no adjustment for risk was applied. Some key elements of the NPV Calculation are described below.

Preclinical cost was set at $10 M over 2 years. This figure includes the cost of development after a product is identified, and covers pharmacokinetics and toxicology studies, pilot formulation work, and minimal drug production required for an IND filing. Clinical costs were based on size and length of studies. Common per patient per year costs were based on study types. The cost of product manufacturing was built into the cost per patient number.

Since the NPV spreadsheet was designed to work in whole years, clinical trial times were averaged to the nearest whole year, rounding up at the halfway point. In order to preserve accuracy of cost prediction over rounded years, the cost estimates during actual trial lengths were spread out over the rounded trial length. Annual overhead was set at $2 M, independent of market size. It should be noted that during Phase 3 and Approval phases, annual overhead includes the cost of continuing to treat patients from previous trials in extension studies. This continued treatment is routinely done in most if not all rare disease studies. In the post-marketing phase, patents would certainly stretch out sales beyond that for orphan drug protection and change the economics. However, at the decision-making stage for investment in rare disease treatments, often no patents are available and compounds are in the public domain so the decision to invest assumes no patents were in existence. During the commercial phase, manufacturing, distribution and marketing costs were estimated at 60% of revenue, and this figure includes post-marketing commitment costs such as product support and patient registries other than the AA- related Phase 4 commitment.

### Treatment cost selection

Intravenous ERT in the case of severe rare diseases was estimated at $300,000 while the cost of intrathecal ERT would approximate $200,000/year, based on current ERT costs (Table 2). Small-molecule therapy for severe diseases was estimated to be $120,000, a similar order of magnitude to miglustat for Gaucher disease type 1 treatment, at about $80,000/year. Mannose for CDG-Ib was estimated at $50,000/year. Companies were not consulted in these pricing estimates, and they are rough estimates provided only for relative comparisons.

### Revenue determination

The estimated number of patients was multiplied by price of therapy in order to calculate estimated revenue. Number of patients was calculated by multiplying incidence rates and average life span. Alternatively, when patients had been directly counted, this value was multiplied by 3, and when there existed a published number of patients described in the literature, the number of reported patients was multiplied by 5 to account for underrepresentation in the literature. To estimate these values, the OMMBID and Orpha.net were searched. There is no precise and accurate way to determine market size in rare diseases.

### Statistical analysis

For all determinations of sample size, a p-value of 0.05 and a power of 80% were used. When endpoint values with standard deviations for both untreated and treated patients, or untreated and control patients, were available, a two sample t-test analysis was performed [41] to calculate sample size. If the percentage of patients experiencing an event or symptom before and after treatment were given, a comparison of event rates between two independent cohorts was done [42]. The result used was the one "assuming outcome data will be analyzed prospectively by Fisher's exact-test or with a continuity corrected chi-squared test [42]." This assumption was chosen based on small clinical trial size. For some programs with surrogate endpoints, the study size estimate based on power calculations would have been too small for reasonable expectations of safety exposure, and in these cases, a minimum study size was applied.

## Results

### Numerous rare diseases exist with successfully treated animal models and stalled clinical development

We searched PubMed to locate publications describing successfully treated animal models with rare disease treatments not yet approved in the US or EU. These treatments were either completely stalled in development, or are currently in the process of development, though delayed. Stem cell and gene therapy treatments were not considered due to the potential complexity of translation to humans, and instead we focused on protein or small molecule therapies. Our experiences, as well as the relative ease of treatment, led us to primarily focus on inherited disorders of metabolism. Endocrine, nutritional, and metabolic disease treatments were found to be among the most likely to have development programs initiated [11]. We identified 15 diseases having a relevant corresponding animal model, a treatment with a known mechanism, and a treatment effect which appeared to be potentially clinically relevant [26,43-56] (Table 1). This disease selection is not intended to be an exhaustive listing of all possible successful treatments in animal models, but represents a sufficient set of examples for study.

### Comparing cost of development using clinical or surrogate endpoints

In order to rigorously investigate the differences between the use of clinical and surrogate endpoints, we first identified reasonable endpoints for our 15 diseases (Table 2). We sought and obtained input from experts to help guide our choice in the determination of both types of endpoints. All endpoints, their values, expected treatment effects, and timeframe needed to detect change can be located in Table 2. Additional details on endpoint selection are in the online Methods section. Although we believe that these endpoints are reasonable, we do not imply that we have singled out the optimal ones, nor that these endpoints currently meet the qualification criteria a regulatory authority might require for use in pivotal clinical study. Our aim is to illustrate the benefit of surrogate endpoint use to demonstrate the potential value and stimulate discussion of appropriate qualification criteria.

**Figure 1:**
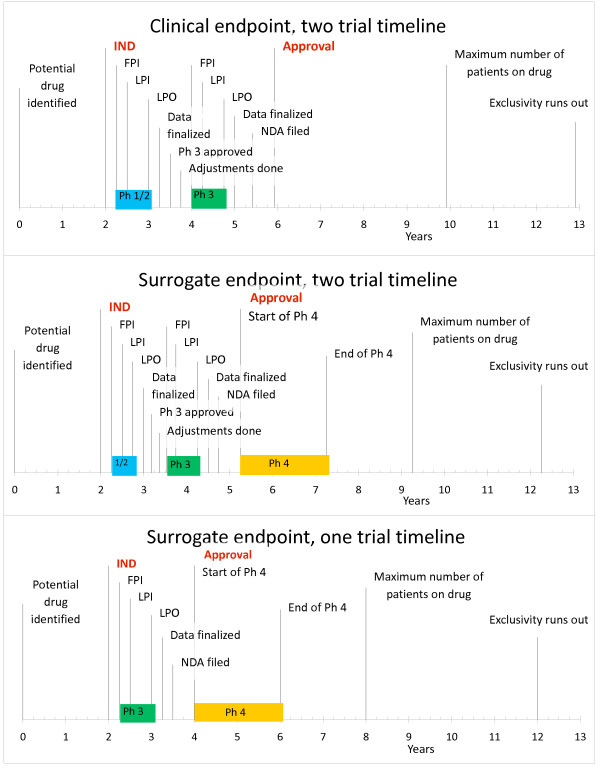
**Three model clinical development programs used to estimate costs**. The top program shows a typical abbreviated two study clinical program as a starting place for analysis. The two study surrogate endpoint study uses the same pre-approval studies but adds a post-approval Phase 4 study. The third program assumes that a single clinical study would be conducted preapproval and that a Phase 4 confirmatory study would be conducted. Timelines begin once a drug has been developed and shown to be effective in an animal model; a 2-year preclinical time period follows, during which pharmacology-toxicology studies and clinical drug production are conducted, at a cost of $5 M/year. Clinical trials then occur with time allotted for study startup, enrollment, and discussion with regulatory authorities, followed by a six month priority review approval process. IND: Investigational New Drug, FPI: First Patient In, LPI: Last Patient In, LPO: Last Patient Out, NDA: New Drug Application.

We constructed three hypothetical development programs using a standardized framework that remained constant in order to facilitate direct comparisons. We then varied clinical trial costs and time, based on each program's unique features and endpoints (Figure 1). To estimate revenue, we established a best estimate of market size based on incidence and likely lifespan (Table 1), and chose market prices for products to be comparable to those for similar approved treatments (Table 2). All hypothetical programs were assumed to be successful at each step, without delays or adverse events, and no adjustments for risk of failure were made. The program costs are therefore conservative and represent a best case scenario.

The first clinical endpoint-based program contains a Phase 1/2 study in which pharmacokinetics and dosing are established and a first look at clinical efficacy occurs, followed by a Phase 3 study; realistic sample sizes and durations for Phase 3 trials were determined based on chosen endpoint properties and historical study sizes. Surrogate-based clinical development programs come in two forms: one surrogate-based program uses a similar 2-study sequence, and a second program uses a single study. Because a surrogate endpoint-based study requires fewer patients and less time to observe treatment effects, the time to enroll patients, treat them, and conduct analyses is shortened. In this analysis, we required a minimum study length and minimum patient enrollment to ensure the collection of sufficient safety data. The 1-study surrogate endpoint-based program has a greater risk of failure because there is less prior clinical experience, less insight into optimal drug dosing, and a lack of data to inform on the choice of endpoints and potential treatment effect size, information which would have been acquired during a Phase 1/2 trial. However, in the case of a disease with an exceptionally small patient population and a dramatic clinical or biochemical effect in the treated corresponding animal model, a single study may be the only financially or clinically viable option. Both surrogate endpoint programs include the cost of an additional 2-year post-marketing study comparable in size and cost to Phase 3, as expected under AA, whereas the clinical endpoint-based program does not. Post-marketing studies are often required even when a drug has been approved under a clinical endpoint-based program. We elected to omit this requirement in our model in order to avoid skewing financial results too favorably toward the surrogate endpoint-based programs. The net present value of a development program, an investment calculation explained below, is reduced by the addition of the cost of a post-marketing study. Our three programs represent increasingly aggressive approaches to drug development and provide us with three points on the cost spectrum.

### Financial impact of surrogate versus clinical endpoint-based clinical development

Clinical development represents both the largest single expenditure and risk factor in achieving a return on an investment. By utilizing either a 2-study or 1-study program using a surrogate endpoint rather than clinical endpoint program, we found that cost to approval decreased by 46% and 62%, respectively (Table 3). The total cost to approval declined from a mean of $90 M to $40 M and $28 M, respectively, representing a savings of $50 M and $62 M over a clinical endpoint-based program. All single-study surrogate-based programs were completed for under $34 M.

**Table 3:** Cost to approval and NPV calculations and analysis for all three clinical development programs.

	Cost to approval			% Decrease in cost to approval		Net present value			
	
Disease	Clinical (all values in millions)	Two-study surrogate	One-study surrogate	From clinical to two-study surrogate	From clinical to one-study surrogate	Clinical	Two-study surrogate	One-study surrogate	Difference between clinical and one-study surrogate
α-Mannosidosis	76	42	28	45%	63%	(29)	(14)	(5)	24
Aspartylglucosaminuria	70	32	23	54%	67%	(0)	17	28	28
Galactosialidosis	76	42	28	45%	63%	(41)	(27)	(19)	22
MPS IVA	85	53	34	37%	60%	188	201	248	59
MPS VII	68	32	23	52%	66%	(24)	(8)	(1)	23
GM1 Gangliosidosis	80	32	23	59%	71%	(16)	9	19	34
LINCL	49	42	28	14%	42%	8	14	28	20
MLD	119	39	28	68%	76%	213	289	342	129
MPS IIIA	119	39	28	68%	76%	19	66	86	67
Niemann-Pick B	56	50	34	10%	39%	39	39	58	19
LAL Deficiency	77	50	34	35%	56%	(35)	(23)	(13)	23
Primary Hyperoxaluria	284	42	28	85%	90%	68	242	290	223
RDEB	105	49	34	54%	68%	(14)	18	33	46
X-Linked HED	57	35	27	39%	53%	(15)	(3)	4	19
CDG-Ib	28	21	17	23%	38%	(21)	(19)	(17)	4
AVERAGE	90	40	28	46%	62%	23	53	72	49

These estimates are for comparison between clinical and surrogate based programs using the NPV spreadsheet in reference 40. The specific value for any program can be substantially different with different assumptions, however for a comparison, these values were considered reasonable based on author experience. In this paper, cost to approval represents total cash invested in a development program after a drug is identified and until the pivotal approval event occurs, when revenue begins. Net present value (NPV) represents a measure of investment return in which the cost of invested capital over time is balanced against the benefit of future revenue, while adjusting for the time value of money. No adjustment for risk of failure was used to avoid having another independent variable complicating interpretation. We chose a standard overhead cost of $2 M/year. We then put in place a 4-year ramp to maximal revenue at 80% of maximum market size. We chose seven years of orphan drug exclusivity, after which we assumed the introduction of a generic that eliminates all further revenue.

**Figure 2:**
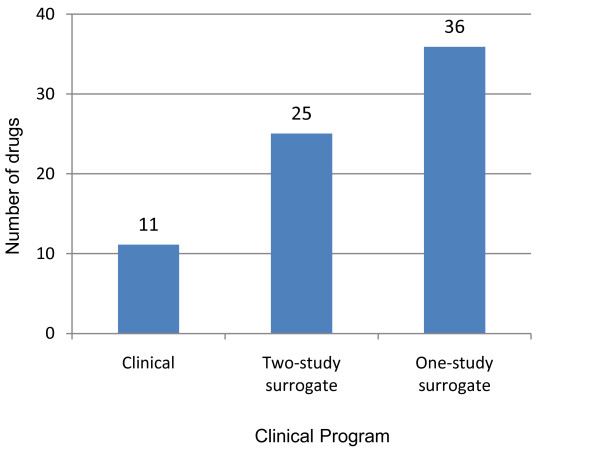
**The number of drugs developed with a $1 billion dollar investment**. We used the cost to launch figures to estimate how many drugs could be developed assuming all programs were successful, and no programs suffered any delays or problems. The comparison is a relative comparison. We did not include any risk factor for the failure of programs, and if they had been added, the surrogate based program number would have been relatively higher than the clinical endpoint driven programs.

The benefit of a decrease in cost to approval is an increase in the number of drugs brought to approval for the same investment (Figure 2). The high cost of clinical endpoint-driven programs results in the development of only about 11 rare disease drugs for an investment of $1B, whereas a 2- or 1-study surrogate-based clinical program would increase this number to 25 and 36 drugs, respectively. Therefore, if we can establish how to manage reasonable AA access, we can triple the number of drugs developed and diseases treated for the same investment.

### Investment value of surrogate endpoint-based programs

Net Present Value (NPV) is the dollar amount that encompasses the overall investment and return for a program, and is commonly used in industry as a tool to assess the value of investing in a program. We did not include an adjustment for the risk of failure, which would have likely skewed results even more favorably toward surrogate endpoint use, which have a greater likelihood for detecting a consistent effect and reducing NPVs in general.

NPVs increase from a mean of $23 M using a clinical endpoint design to $53 M and $72 M using a 2- and 1-study surrogate endpoint design, respectively (Table 3). The most significant gains were observed with a 1-study surrogate compared to a clinical surrogate program. Fourteen out of 15 NPVs increased by $19 M or more. Four out of nine which displayed negative values in a clinical endpoint program changed to positive. Of the remaining six NPVs, three increased by two-fold or better with the AA pathway.

### Case examples

MPS VII is a lysosomal storage disease and one of the rarest of our 15 disorders, with only an estimated 200 patients in industrialized nations. The first successful demonstration of ERT in MPS VII mice showed a significant reduction in accumulation of lysosomal storage in 1994 [52] and many additional papers have since appeared demonstrating successful enzyme therapy in MPS VII animals. Three similar MPS disorders, MPS I, II, and VI, all have approved ERTs, confirming the likely success of a program for MPS VII. The diseases α-mannosidosis, aspartylglucosaminuria, and galactosialidosis are similar cases. Switching from the 6MWT to a urinary storage marker, the needed number of Phase 3 patients is reduced by at least half, a key consideration given the rarity of these diseases, and the total time for development is likely shortened since a broader spectrum of patients qualifies for enrollment. The NPV for ERT in MPS VII is negative for all three models. However, it approaches a positive value in the 1-study surrogate endpoint model.

Primary hyperoxaluria results from overproduction of urine oxalate, leading to kidney stones, loss of kidney function, and oxalosis, the deposition of calcium oxalate crystals in the organs. Mice displaying symptoms similar to primary hyperoxaluria type 1 were treated with an oxalate-degrading enzyme and exhibited histologically normal kidneys with no oxalate crystals and normal kidney filtration [57]. In order to detect efficacy by measuring rates of renal failure, the disease's most damaging feature, a two-year study enrolling approximately 183 patients selected for their particular stage of disease where renal failure is progressing would likely take a long time to enroll and conduct. In contrast, reduction of urinary oxalate is a straightforward measurement that could take as little as three months and 20 patients to detect. Far from being an unreliable marker, excessively high oxalate levels are known to directly damage kidneys. In this case, an NPV of $68 M jumps to $242 M when switching from a clinical endpoint to a 2-study surrogate endpoint model.

Recessive dystrophic epidermolysis bullosa (RDEB) is a disease caused by defects in type VII collagen protein which binds the dermis to the epidermis. Blistering and sores readily occur, open wounds are slow to heal and susceptible to infection, esophageal scarring can cause eating difficulties, and chronic inflammation increases risk of squamous cell carcinoma. Injection of type VII collagen in DEB mice corrected the disease phenotype by decreasing skin fragility, blistering, and prolonging lifespan [58]. Clinical endpoints might include the rate of hospitalizations or skin infections, though these endpoints may be confounded by varying clinical practice or quantities of supportive home care. In contrast, using a measure of skin adhesion, such as number of anchoring fibrils, or a tension test that would measure adhesion strength between dermis and epidermis, would be a more direct measure of drug effect. The NPV for type VII collagen treatment increases from -$14 M to $18 M and $33 M using the 2-study and 1-study surrogate endpoint model, respectively.

The potential benefit of surrogate markers is particularly critical in neurologic disorders. To detect efficacy of ERT in the neurological lysosomal storage diseases, LINCL, MLD, and MPS IIIA, we selected CSF markers for abnormal brain substrate storage and/or brain injury as surrogate endpoints since they should represent the direct adverse impact of storage on brain pathology. Variability in disease expression, the plasticity and complexity of the nervous system, and the indeterminate degree of irreversibility make using neurological clinical endpoints, such as development quotient, challenging. A surrogate marker of neurologic injury or substrate accumulation may provide better evidence of the biochemical effect of treatment in neurologically heterogeneous patients and be a more reliable way to assess treatment benefit.

## Discussion

We have located a number of potential treatments for rare genetic diseases that have yet to be translated to clinical use due to a number of barriers in development. By assessing development costs in model development programs using clinical or surrogate endpoints, we have demonstrated the potential benefit of increased accessibility to the AA pathway in obtaining rare disease drug approval. The use of surrogate endpoints brings about profound changes in cost to approval and NPV. AA accessibility potentially increases the number of drugs developed for rare diseases 3-fold for the same investment, resulting in many more diseases and patients treated in a more innovative and productive development system.

Although concern has been raised that orphan product costs are very high, and that increasing the approval rate of drugs might increase health care costs, we believe that many of these treatments could reduce some clinical care costs as well and reduce the economic impact on families. In addition, as long as development costs are extremely high, the costs of drugs will be driven to stay equally high. By controlling high development costs through improved access to AA, orphan drug cost management is more plausible, without shutting down the incentive for investment in development. In no case should it be said that blocking the development of life-saving, life-changing treatments is an ethical option to save money on healthcare.

This paper does not aim to prove the merit of the proposed surrogates, but only shows how the use of surrogate endpoints could impact investment potential. Improved access to AA depends on whether the surrogate can be shown to be "reasonably likely to predict clinical benefit" [3] by FDA standards. Biotechnology companies have encountered development difficulties because of the lack of clear qualification criteria for surrogate endpoints due to the belief that each must be evaluated on a case-by-case basis. This unpredictable process significantly hinders the initiation of development of many programs that might require a novel surrogate endpoint. As an example, the use of the surrogate endpoint of kidney biopsy to gain approval for the treatment of Fabry was initially resisted by FDA, which sought greater assurance that the pathologic surrogate was predictive of clinically meaningful benefit. The renal pathology marker for Fabry was eventually affirmed by the Metabolic and Endocrine Advisory Committee Meeting in January 2003 [59] and over time, has been shown to predict clinical outcome in a confirmatory study. Other examples exist of surrogate endpoints in rare diseases that have substantial animal model data to support them, but are rejected due to regulatory uncertainty of their ability to predict clinical outcome [7,8]. Some uncertainty for surrogates will always exist in rare diseases with limited patient-based clinical data. The creation of a precise guidance or established criteria to support researchers, those involved in treatment development for rare diseases, and investors would help foster the effective use of appropriate surrogates and improve access to the AA pathway.

The use of surrogate endpoints poses a risk for the approval of ineffective or unexpectedly harmful treatments. This concern has heightened due to recent high profile failures of large market drugs. Some surrogates were unable to provide accurate assessments of clinical benefit or failed to capture adverse effects unrelated to the drug's mechanism of action, as in the case of cardiac arrhythmias or many other diseases [60]. For instance, encainide and fleicanide were successful in suppressing arrhythmia, with the intent of decreasing risk of sudden cardiac death, but were later found to bring about a 3-fold increase in sudden cardiac death [61]. However, such cases may not provide an applicable cautionary example of surrogate use for many life-threatening rare diseases because many rare biochemical disorders have far more relevant and direct biochemical or pathologic markers. These biomarkers are directly related to the disease pathophysiology and also to the mechanism of drug action, and so should more accurately assess whether a drug has a beneficial treatment effect. This holds true even if the ability of the endpoint to predict clinical outcome is yet unproven. It is plausible that some of these drugs may not demonstrate clinical efficacy in Phase 4, but given that the drugs should be reasonably safe through the required clinical evaluation, we believe that families will accept the possibility that the drugs' efficacy may be less than expected. To date, no rare disease drugs approved on biochemical endpoints have been withdrawn for lack of efficacy or safety issues. Our analyses do require minimum patient exposure in clinical trials to ensure a reasonable degree of safety, even if efficacy using the surrogate endpoint could be proven with smaller patient numbers.

Surrogate endpoints are not simply a convenience, but are a necessary part of the development path for some ultra-rare disorders. Surrogate endpoints may be indispensable for clinical trials when too few patients exist to conduct a large clinical endpoint-driven, double-blind trial. Diseases with substantial heterogeneity or variable irreversibility of disease symptoms, such as bone or neurologic disease, may also require surrogate endpoint-driven trials to ever have the chance to be treated. It is difficult to detect drug efficacy in treating diseases manifested by a variety of symptoms when not every patient expresses each symptom; those who are not affected in one aspect of a disease cannot, with therapy, show substantial improvement in that aspect, even if overall improvement in health is significant. It is also difficult to detect drug efficacy in treating diseases with varying levels of irreversibility, which results when a disease is diagnosed and treated after chronic tissue damage has occurred. If a patient has been permanently changed by disease, a small clinical improvement may appear to be a failure, even if the degree of improvement was the maximum possible for that patient. Surrogate endpoints may be more effective than clinical endpoints in detecting efficacy in these situations because they can indicate that the drug is having the correct metabolic reversal effect regardless of the reversibility of the accumulated clinical disease symptoms. Since patients with some diseases such as neurologic disorders are commonly diagnosed too late in their disease course for effective disease reversal, the biochemical treatment effect may be the only readily measurable benefit. Once a drug is approved, there will be a strong incentive to diagnose patients earlier in their disease course as a result of the availability of treatment [62]. Clinical benefit must then be verified over time by studying early-treated patients in post-marketing studies over several years. With these development steps using the AA pathway, a drug can be developed and the natural history of the rare disease is forever changed by the approved treatment. This development course to early rare disease management can never be initiated if the first step to treatment approval via the AA pathway is not taken.

We cannot and should not expect surrogates to accurately and quantitatively predict all clinical outcomes. The relationship between a blood or urine test and a clinical outcome will rarely be a perfect linear proportional relationship, nor can a surrogate endpoint quantitatively predict the complex interplay of severity, progression, and other clinical factors on a given clinical outcome. The surrogate can provide a clear indication that the treatment effect is occurring and that the probabilistic outcome of clinical benefit is substantially improved. A reasonable degree of predictive value and risk must be determined, based on sound science, to allow therapies to begin the path to development. Discussion between patient groups, biotechnology leaders, drug developers, and regulatory authorities is essential to develop clear criteria for rare or ultra-rare diseases, detailing necessary features of a usable surrogate endpoint that will protect patients and guide early research toward providing the needed information. Clear criteria are also essential to biotechnology companies and investors since only a high degree of certainty regarding the feasibility of a surrogate-endpoint driven pivotal study will increase the incentive to initiate development programs in many rare diseases. If a reasonable guidance were provided which defines scientifically-sound surrogates and achievable qualification criteria for the AA pathway to biotechnology companies, investors, and patients alike, rare disease drug development could potentially grow many fold as renewed biotechnology investment becomes plausible. Difficult and tragic diseases will then finally have a chance to be treated based on cutting-edge science.

## Conclusion

By highlighting fifteen ultra-rare disease examples, we have demonstrated the existence of promising, scientifically-sound animal model therapies that have not yet been translated to the clinic. We evaluated clinical- or surrogate-based clinical development programs for these fifteen diseases and showed that the use of the AA pathway led to a decrease in cost to approval of 62%, and an increase in NPV from a mean of $23 M to $72 M. For the same biotechnology company investment, three times as many ultra-rare disease drugs could be developed with AA. Collaboration between academia, government, and industry is essential in order to set clear and practical regulatory criteria for use of AA in ultra-rare disease treatment development, accelerating the translation of science into treatments for ultra-rare disease patients.

## Competing interests

EK was an executive officer and has income related to Aldurazyme treatment for MPS I, and currently is chief executive officer of a new company focused on ultra-rare treatment development and owns stock or receives income from these entities. In these capacities, he has been or is directly involved in treatment development of some of the diseases discussed. BM has no competing interests.

## Authors' contributions

BM conducted the literature research and analyses, and wrote the drafts. EK designed the research project, oversaw the choice of diseases, endpoints, study designs, cost estimates, NPV calculations and edited the manuscript. Both authors read and approved the final manuscript.
